# Experiences of ethnic minority patients who are living with a primary chronic bowel condition: a systematic scoping review with narrative synthesis

**DOI:** 10.1186/s12876-021-01857-8

**Published:** 2021-08-18

**Authors:** Salina Ahmed, Paul D. Newton, Omorogieva Ojo, Lesley Dibley

**Affiliations:** School of Health Sciences, University of Greenwich, Southwood Site, Avery Hill Road, Eltham, London, SE9 2UG UK

**Keywords:** Coeliac Disease, Crohn’s Disease, Ethnic minority, Experiences, Inflammatory Bowel Disease, Irritable Bowel Syndrome, Systematic scoping review, Ulcerative Colitis

## Abstract

**Background:**

Prevalence of chronic gastrointestinal diseases has been rising amongst ethnic minority populations in Western countries, despite the first-generation migrants originating from countries of low prevalence. Differences caused by genetic, environmental, cultural, and religious factors in each context may contribute towards shaping experiences of ethnic minority individuals living with primary bowel conditions. This review aimed to explore the experiences of ethnic minority patients living with chronic bowel conditions.

**Methods:**

We conducted a systematic scoping review to retrieve qualitative, quantitative, and mixed methods studies from eight electronic databases, and manually searched reference lists of frequently cited papers.

**Results:**

Fourteen papers met the inclusion criteria: focussing on inflammatory bowel disease, irritable bowel syndrome, and coeliac disease. Core themes were narratively analysed. South Asians had limited understanding of inflammatory bowel disease and coeliac disease, hindered by language and literacy barriers, particularly for older generations, suggesting that culturally relevant information is needed. Family support was limited, and Muslim South Asians referred to religion to understand and self-manage inflammatory bowel disease. Ethnic minority groups across countries experienced: poor dietary intake for coeliac disease and inflammatory bowel disease, cultural conflict in self-managing diet for inflammatory bowel disease which increased anxiety, and there was a need for better quality of, and access to, healthcare services. British ethnic minority groups experienced difficulties with IBD diagnosis/misdiagnosis.

**Conclusions:**

Cultural, religious, and social contexts, together with language barriers and limited health literacy influenced experiences of health inequalities for ethnic minority patients living with chronic bowel diseases.

**Supplementary Information:**

The online version contains supplementary material available at 10.1186/s12876-021-01857-8.

## Background

Chronic bowel illnesses have been rising in ethnic minority populations, though experiences of patients in ethnic minority groups have been largely underexplored [1–5]. Common bowel conditions include Inflammatory Bowel Disease (IBD): an umbrella term that covers various conditions causing gastrointestinal inflammation including Crohn’s Disease (CD) and Ulcerative Colitis (UC); Irritable Bowel Disease (IBS): where recurrent abdominal pain/discomfort has an impact on defecation and changes in bowel habits, but physiological changes to confirm diagnosis are absent [6–8]; and coeliac disease: an auto-immune condition arising from a dysregulated immune response to gluten (a storage protein found in wheat, barley, and rye) in the diet, where the reaction to gluten damages the enteral villi causing shortening and blunting which decreases the surface area of the mucosa and thus affects absorption of nutrients from the gut [9]. All three bowel conditions are characterised by symptoms of abdominal pain, rectal bleeding, fatigue, urgency, and diarrhoea, and are usually diagnosed via colonoscopy and/or endoscopy [8, 9].

Earlier studies have shown that IBD prevalence has been geographically distributed in Northern/Western European, North American, and Australian White populations [10–12]. Similar trends have also been observed in North American and Western European White populations for IBS [4, 5, 13], and across White populations in Europe for coeliac disease [9, 14]. Those who have migrated from low prevalence countries (e.g., South Asia) to high prevalence countries (e.g., UK) and their offspring, seem to have higher rates of some chronic bowel conditions than the indigenous population including IBD [15–19] and coeliac disease [14]. Genetic susceptibility, and cultural and environmental influences may play a part in shaping the diverse experiences of ethnic minority populations, raising important implications for disease management and intervention development for these communities, which need to be better understood [20–24].

Byron et al. [22] framed experiences of chronic bowel illnesses as distinct daily ‘challenges’ that increase disease activity, whether physical (e.g., fatigue) and/or psychosocial (e.g., anxiety), and reasoned that people adapt to self-manage these challenges (e.g., awareness of and being proximal to bathroom facilities), although little is known about the challenges that exist in ethnic minority groups. Dietary changes in these groups can be affected by migration (e.g., accessibility and the availability of traditional diets or ingredients such as fresh fish) and acculturation (e.g., dietary modifications to assimilate with food choices of indigenous groups such as processed food), which may affect the gut microbiome [1, 15–17, 25]. To illustrate this, Limdi et al. [26] found significant differences between British South Asians and White patients’ beliefs, perceptions, and behaviours around IBD and dieting/food avoidance, even though South Asians comprised of a small cohort. More South Asians restricted their diet to avoid relapses and eating outdoors. They had a greater belief that diet contributed to disease initiation and controlled IBD better than medication. Similar findings were also reported in another UK based study with a larger number of participants [27]. A Gluten Free Diet (GFD) can be an effective but challenging way of managing coeliac disease, since a GFD necessitates pre-existing knowledge or access to information, motivation, and availability of GF food, and avoidance of habitual/traditional gluten heavy food (e.g., chapati for Punjabis) requires cultural advice [14, 28, 29]. Adam et al. [30] found significant differences in adherence to GFD (64.6% vs. 12.1%) and vitamin D deficiency (70.8% vs. 32.8%) between British Caucasians and South Asians, despite the latter group comprising of a smaller cohort.

Heterogeneity in disease phenotype in ethnic minority groups can be influenced by differential cultural and environmental exposures (e.g., pollutants, smoking, and microbial exposure) forming a generational impact [15, 31]. Misra et al. [31] found that heightened genetic susceptibility and environmental triggers may have promoted the risk of developing UC with a non-colonic phenotype for British Indian patients, and most second-generation Indians (aged 15–40) had higher age-adjusted incidence of UC compared to White Europeans and Pakistanis, indicating that subcultural differences amongst South Asians themselves needs further consideration. Carr and Mayberry [15] found greater disease severity in second generation South Asians with UC (living in the UK for at least 25 years) compared to first generation migrants and the indigenous population, although the reason for such differences remain unclear. There may also be differences in healthcare services received by ethnic minority patients [24, 31, 32]. Silvernale et al. [32] found that ethnic minority groups (Hispanics, Blacks, Asians) with IBS were significantly less likely to receive consultation appointments in secondary care compared to White patients, but they were more likely to receive gastroenterology procedures (e.g., diagnostic testing) compared to White patients. Similarly, Misra et al. [31] found that biological therapy for CD was prescribed significantly less often for British South Asians compared to White patients.

We aimed to conduct a systematic scoping literature review of ethnic minority peoples’ experiences of living with chronic bowel diseases, including IBD, IBS and coeliac disease. The purpose was to examine the available evidence, identify gaps in the literature and outline, appraise and synthesise all relevant studies rather than generate a definitive answer to a specific question [33, 34]. A broader view was taken since a preliminary literature search identified insufficient evidence for a classic systematic review addressing only IBD or IBS amongst this patient population.

## Methods

We followed the systematic scoping review process described originally by Arksey and O’Malley [35] and developed more recently by Pollock et al. [34]. The process mirrors that of a classic review but allows adjustment to the protocol as a sense of the literature emerges, and quality appraisal is optional. Both factors are methodologically sound because the purpose is not to provide a precise answer to immediately inform practice or policy, but to identify the types of available evidence, and identify and analyse any knowledge gaps [34]. Conclusions may therefore be broader than expected from a classic systematic review. However, we did conduct a quality appraisal as a further purpose of this review was to inform the design of our intended future studies.

### Literature search

Search terms were finalised using the Sample, Phenomenon of Interest, Design, Evaluation and Research type (SPIDER) framework (see Table 1 and Additional file 1: Appendix S1) [36]. The SPIDER framework [36] is a systematised search strategy tool that facilitates rigour and confidence in the retrieval of studies in a review, similar to the PICO [37], though the SPIDER framework allows more flexibility for considering various study designs (e.g., qualitative and mixed methods research) than PICO which is suited specifically for quantitative studies [36, 37]. We searched for qualitative, quantitative, and mixed methods studies on eight electronic databases (CINAHL, PubMed, PsychINFO, Psychology and Behavioural Sciences Collection, Ovid, Embase, Academic Search Primer, and Google Scholar), and manually searched reference lists of frequently cited papers. The search was limited to articles published in the English language, since 2000, reflecting the timeframe for significant developments in medical interventions in conditions such as IBD in the last 20 years [38, 39], and the rising incidence of primary bowel conditions in ethnic minority populations [1–3].

**Table 1:** Search terms based on the SPIDER framework

SPIDER framework	Search terms
Sample (S)	Ethnic* minorit* OR Indigenous OR Native
	South Asia* OR Bengali OR Bangladesh* OR India* OR Pakistan* OR Sri Lanka* OR Nepal* OR Afghan* OR Black OR Africa* OR Afro Caribbean OR Somali OR Sudan* OR Zimbabw* OR Turk* OR Arab OR Middle Eastern OR Assyrian OR Kurd OR Iraq* OR Leban* OR Syria* OR Iran* OR Irish OR Gypsy OR Traveller OR Refugee OR Asylum OR Chin* OR Japan* OR Korea* OR Mongolia* OR Latin* OR Puerto Rican OR Mexic* OR Hawai’ian OR Alaska OR Brazil* OR Chile* OR Venezuelan OR Jamaica* OR Cuban OR Hispanic OR Quebec* OR Mohawk OR Inuit OR Metis OR Acadian OR Aboriginal or Melanesian or Maori or Islander OR Filipino OR Indonesia* OR Vietnam* OR Cambodia* OR Burm* OR Malaysia* OR Singapore* OR Timorese OR Laotians OR Europe*
Phenomenon of Interest (PI)	Inflammatory bowel disease OR Crohn OR Ulcerative colitis
	Bowel function OR bowel dysfunction OR bowel disorder OR bowel cancer OR Irritable bowel syndrome
	Carcinoma OR constipation OR stoma
Design (D)	Interview OR focus group OR survey OR case stud* OR observation OR ethnograph*
Evaluation (E)	Experien* OR view* OR opinion* OR attitude* OR feel* OR understanding* OR belief* OR perspectives* OR perception*
Research type (R)	Qualitative OR quantitative OR mixed method

### Inclusion and exclusion criteria

We conducted a preliminary scan of the literature to get a sense of the data and based our inclusion criteria on this. The search included people of all ages and studies that were: 1) full text original research articles; 2) published in English, since 2000; 3) comprised of all ethnic minority participants (as described by authors) or studies reporting findings of ethnic minority participants separately from non-minority group participants; 4) participants who were resident of countries such as the UK, USA, Australia, and New Zealand; 5) participants living with any primary chronic bowel condition; 6) any qualitative, quantitative, or mixed methods design. We excluded studies that: 1) did not clearly describe participant ethnicity e.g., non-white; 2) the bowel condition or symptom is not explicitly linked to patients’ experiences; 3) experiences of carers, parents, or healthcare professionals; 4) studies on international travellers who are not resident of a country.

### Study selection

**Fig. 1:**
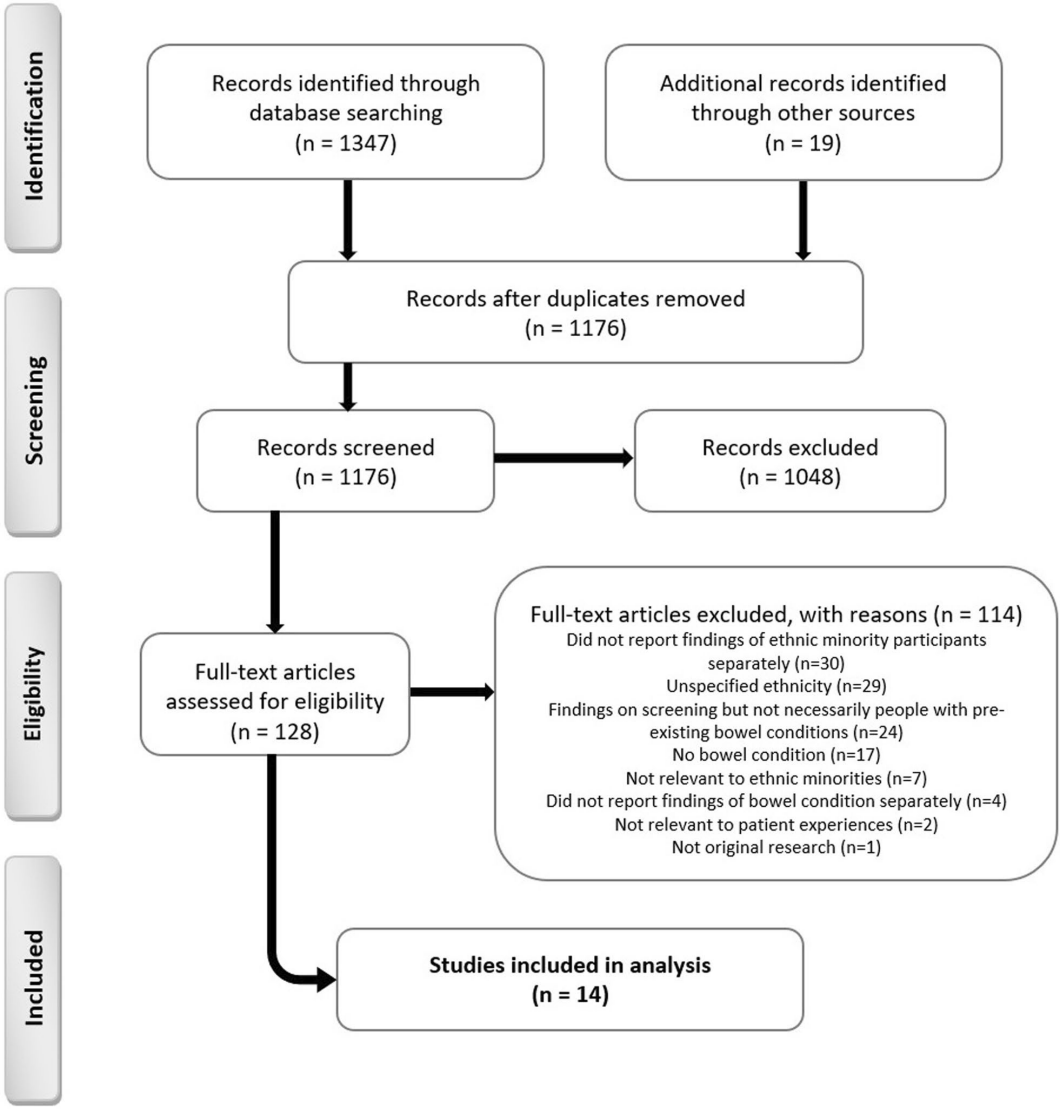
PRISMA flow diagram

A PRISMA diagram was used to report the application of the inclusion and exclusion criteria and the selection of final papers for review (Fig. 1).

### Data extraction, quality assessment and analysis

All titles, abstracts and full texts were screened by one reviewer (SA); in addition, a random 10% of titles and abstracts were screened by two other reviewers (PN, OO), and 10% of full texts were screened by three reviewers (PN, OO, LD). Disagreements were resolved by discussions and clarification of the inclusion/exclusion criteria as needed. Two recognised critical appraisal tools were used to critique each included paper and informed data extraction: The Centre for Evidence-Based Management Critical Appraisal (CEBMa) for survey-based studies [40], and the Critical Appraisal Skills Programme (CASP) for qualitative, case-controlled and cohort studies [41]. Quality appraisal was reviewed by two reviewers (SA, OO). Due to heterogeneity of designs across studies, a data-driven approach to thematic analysis was taken to identify core themes of patient experiences and this was summarised narratively [42].

## Results

### Study characteristics

Of the 1347 articles that were originally retrieved, 14 papers were retained for inclusion in the review following screening (Fig. 1). Table 2 illustrates that studies were conducted between 2001 and 2018. Seven studies were conducted in the UK [43–49], six in the USA [50–55], and one in Malaysia [56]. Study design varied and included six case-controlled studies [46, 47, 50, 51, 53, 55], four survey-based studies [44, 45, 52, 54], three qualitative studies [43, 48, 49] and one cohort study [56]; focussing on IBD [43, 45–53, 55], IBS [54, 56], and coeliac disease [44]. Studies recruited participants from various ethnic minority groups including South Asians, African Americans, Blacks, Hispanics, and Chinese. Three studies that mentioned religious backgrounds comprised of a mixture of religions, where the majority of participants were Muslims [43, 48, 49]. Thirteen studies indicated participants’ ages; three used the inclusion criteria to specify participant age as 16–24 [43, 46, 49]; one study reports participant age as over 16 [44]; another study reports participant age as 18–65 [48]; and nine studies report mean ages with one standard deviation in the results enabling the reader to determine that all participants were over the age of 16 [47, 50–54, 56]. The remaining two studies [45, 55] give no indication of participants’ ages. Five studies accommodated for language alongside English, which were mainly South Asian languages (e.g., Hindi, Gujrati, Punjabi, Bengali, Urdu, and Mirpuri) [44–46, 48] and Spanish [50].

**Table 2:** Summary of study characteristics

References Country	Population 1. Disease focus 2. Sample size 3. Ethnicity 4. Age/gender 5. Other e.g., language, religion	Design 1. Design 2. Method 3. Sampling method 4. Setting	Outcome measures/instruments a. Psychological health b. Quality of life c. Physical health d. Social health e. Miscellaneous	Main results	Strengths/limitations	Quality rating
*Inflammatory Bowel disease*						
Alexakis et al. [43] UK	1. CD (13); UC (6); Other (1) 2. 20 patients 3. South Asian (17; 7 Pakistani, 5 Bangladeshi, 3 Indian, 1 Sri Lankan, 1 Nepal); Mixed white/Asian (1), Black (2) 4. 16-24; male (13); female (7) 5. English; Muslims (13), Christian (2), Hindu/Buddhist (2), Other/Agnostic (2), Mormon (1),	1. Qualitative 2. Interviews 3. Nil; appears purposive 4. Three gastroenterology clinics	e. Semi-structured interview to explore experiences	Culturally appropriate IBD information was needed for South Asian parents who had low awareness due to factors such as language barriers. Young people also experienced tensions between effective self-management strategies (e.g., dieting, and religious coping) and cultural norms and practices e.g., spicy food and interruption of prayer for bowel movements. Common experiences across ethnicities included difficulty attaining diagnosis/misdiagnosis and experiencing poor support from primary care services and the educational system.	Detailed data; data saturation; pilot study/small sample for generalisation; ethnic mismatch of interviewer and interviewee	CASP 4*
Conroy and Mayberry [45] UK	1. UC 2. 56 patients 3. South Asians 4. Nil; nil 5. English, Hindi, Gujarati, Punjabi	1. Survey-based 2. Survey; questionnaire and telephone/post contact 3. Not stated; appears purposive 4. Healthcare IBD register	e. Postal survey measured initial demand for UC information; follow-up questionnaire measured UC information needs; follow-up telephone and post contact to encourage a good response rate	There was a need for English information on UC to be translated into South Asian languages. Majority of patients provided positive feedback for leaflets offered in English, Hindi, Gujarati, and Punjabi. All patients found the leaflets useful.	Language accommodation/low response rates	CEBMa 3*
Damas et al. [50] USA	1. IBD including CD (12) and UC (17) 2. 58 (29 IBD patients, 29 non-IBD control) 3. Hispanics—Cuban, Colombian or Other Latin American 4. Over 18; male (11 control, 14 IBD) 5. English, Spanish	1. Cross-sectional case-controlled 2. Questionnaires 3. Not stated; appears purposive 4. University-based gastroenterology clinics	e. Abbreviated Stephenson Multi-Group Acculturation Scale e. A 24-h diet recall (the ASA-24) e. The Healthy Eating Index (HEI-2010) measured diet quality	Hispanics were mainly bicultural. Most changed their diet after immigration. Patients and controls had poor eating habits, irrespective of the presence of IBD e.g., lower than recommended consumption of total vegetables, legumes, whole grains, and sea plant protein. Compared to controls, non-control patients reported lower intake across all items, particularly total fruit, whole fruit, and total dairy. Non-control IBD patients had higher consumption of sodium, refined grains, and empty calories e.g., alcohol, solid fats and added sugars.	Representative sample from different countries/small sample size; daily nutrition may differ depending on disease activity	CASP 3*
Farrukh and Mayberry [46] UK	1. UC 2. 70 patients 3. South Asian (28), White European (42) 4. 16-24; male (16 South Asian, 22 European); female (12 South Asian, 20 European) 5. English and South Asian (unspecified)	1. Retrospective case-controlled 2. Cases 3. Not stated; appears purposive/convenience 4. Three hospitals and community hospital notes	c. Record case notes of 1996–1998 and clinical records measured clinical outcomes on surgery and deaths	Surgical and death rates were the same for both South Asians and White Europeans. However, there were differences in provision of care, where South Asians received poorer quality clinical care than Europeans e.g., South Asians were significantly less likely to see a consultant and more likely to be discharged. South Asians were hospitalised more often but had significantly fewer tests than European patients. More screening colonoscopies were offered to Europeans than South Asians, although non-significant.	/Retrospective analysis and 29% of case records have been destroyed, were incomplete or could not be retrieved	CASP 3*
Jackson et al. [51], USA	1. CD 2. 99 patients 3. African American (55); Whites (44) 4. mean age 32 White, 30 African American; male (50; 26 African American, 24 white); female (49; 29 African American, 20 white) 5. Nil	1. Retrospective case-controlled 2. Interviews; cases; survey 3. Not stated; appears purposive/convenience 4. Three gastroenterology clinics	c. Telephone interviews; chart reviews; standard evaluations; surveys, measured disease location, surgery, and medication use c. Patient statements measured compliance c. Clinician assessment measured CD diagnosis	CD may be different in African Americans compared to White patients: small bowel disease and small bowel resection was more frequent in White patients. Colonic disease and perirectal fistulae were more frequent in African Americans. White patients sought care for their CD in a clinic setting and reported greater/complete compliance with medical therapy. African Americans more frequently discontinued medical therapy. Both groups felt equally informed about CD, but a greater percentage of White patients felt that their disease was under good control.	/Most African Americans were recruited from a hospital with costly medications	CASP 4*
Goodhand et al. [47], UK	1. IBD 2. 238 patients 3. Bangladeshi (119); White Caucasian (119) 4. Mean (SEM) 29.6 (1.1) Bangladeshi, 30.9 (1.1) White; male (72 Bangladeshi; 52 White Caucasian); female (47 Bangladeshi, 67 White Caucasians) 5. Nil	1. Retrospective case-controlled 2. Cases 3. Case-based 4. IBD outpatient clinic	c. Electronic patient record and IBD database measured demographic data on place of birth and year of migration d. Online Acorn database measured socio-economic data	There were no differences in adjusted age at diagnosis of IBD between Bangladeshis and White Caucasians. Compared to Caucasians, more Bangladeshis were diagnosed with CD than UC. Crohn’s phenotype at diagnosis was similar in both groups. But Bangladeshis developed perianal complications and received anti-TNFs earlier and underwent surgery later than White Caucasians. More Bangladeshis with UC had extensive disease, and were anaemic and vitamin D deficient, compared to Caucasians.	Bias avoided by matching diagnosis age; accurate phenotyping in single cohort/interpretation and missing data bias; small sample size	CASP 4*
Mukherjee et al. [48], UK	1. IBD including CD (18) and UC 14 and unclear (1) 2. 33 patients 3. South Asians (20 Indian, 9 Pakistani, 4 Bangladeshi) 4. 18-65; male (13); female (20) 5. Bengali, Gujarati, Hindi, Punjabi, Urdu, Mirpuri, English; Muslim (16), Hindu (9), Sikh (7), no faith (1)	1. Qualitative 2. Interviews 3. Purposive 4. Five gastroenterology clinics	e. Telephone and face-to-face interviews to explore experiences and met and unmet need for support	IBD experience influenced by South Asian culture; low awareness meant that the community had difficulty understanding IBD, and religion; difficulties performing ablution and praying. Mostly positive experiences of gastroenterology services, though there was a focus on medical treatment and language barriers existed. There was an emotional toll (e.g., anxiety) that influenced involvement in activities when symptom free. Practical and emotional support was missing in immediate and extended family. Majority adhered to prescribed medication, but also used complementary and alternative medicine.	Representation of South Asians; interviewer living with IBD/time and resources; people over 65 were not recruited; mostly English-speaking participants	CASP 4*
Nash et al. [49] UK	1. CD (13); UC (6); CD and UC (1) 2. 20 patients 3. South Asians (17); Mixed Asian and White (1); Black (2) 4. 17–24; male (13); female (7) 5. English; Islam (13); Christian (2); Agnostic/No religion (2); Hindu (1); Hindu/Buddhist (1); Mormon (1)	1. Qualitative 2. interviews 3. Opportunistic/convenience 4. Three hospitals	e. Telephone and face-to-face interviews measured social inclusion and experiences	Experiences influenced by sociocultural factors such as culture; conflict of diet choices from low awareness of South Asian parents who have English as a second language, and religion; Islamic religious self-management was beneficial. Need for culturally appropriate information for parents, support from schools (mentoring, peer support) and healthcare professionals (communicating information to parents), and family counselling. There were both positive and negative healthcare service encounters. Generic experience across ethnicities included secrecy about IBD, social isolation, diagnosis delays/misdiagnosis and disruptions to education.	Rich data; data saturation/generalisation to the local population	CASP 4*
Nguyen et al. [52] USA	1. IBD including CD and UC 2. 235 patients 3. Black (120); White (115) 4. over 18/nil 5. English	1. Cross-sectional survey-based component of a longitudinal study 2. Telephone questionnaire 3. Purposive 4. IBD outpatient clinic	b. SIBDQ measured QOL c. Chapel Hill Index/Simple Colitis Clinical Activity Index measured disease severity c. HBCS measured medication adherence and appointments d. TIPS	Overall adherence was 65% for both ethnicities. Higher adherence correlated with greater trust-in physician, increasing age and worsening health-related QOL. Adherence was also higher among White patients compared to Black patients. Trust-in-physician, race, and age remained predictors of adherence to medical management after adjustment for employment, income, health insurance, marital and socioeconomic status, and immunomodulator therapy.	Adherence measured during asymptomatic periods/all White physicians; potential bias in oversampling older patients; self-reported data	CEBMa 3*
Strauss et al. [53] USA	1. CD 2. 552 patients 3. Black (145); White (407) 4. Mean age 39 Black, 44 White; male (29 Black); female (37 white) 5. English	1. Case-controlled 2. Survey 3. Convenience 4. Four teaching hospitals and five private practices	b. Medical Outcomes Study Short Form 36 (SF-36) Health Survey measured health status and functioning c. CD aActivity Index (CDAI); the Chapel Hill Index disease activity c. Survey on demographic, surgical and hospitalisation data, medication/compliance history, healthcare utilisation/access	Both ethnicities have similar disease presentation and course e.g., age of CD onset, lag in diagnosis time, frequency of gastroenterology-related hospitalisations and surgeries, and medication use. However, there were also differences: Black patients had lower QOL for all categories compared to Whites. White patients were more likely to have health insurance and could identify a regular provider compared to Black patients	Representative sample; good sample size/recall and information bias; generalisability of findings outside tertiary care hospitals	CASP 4*
Yarur et al. [55] USA	1. IBD including CD and UC 2. 142 patients 3. Hispanic (67; 2 Black), non-Hispanic (75; 17 Black) 4. Nil; male (74); female (24 Hispanic, 43 non-Hispanic) 5. Nil	1. Case-controlled 2. Cases 3. Case-based 4. IBD outpatient clinic	c. Inpatient and outpatient medical records measured predictive pre-operative variables and post-operative outcomes	A small increase in post-operative complications in Hispanics compared to non-Hispanics with equal access to medical care and follow-up, but this did not reach significance. Factors independently associated with postoperative complications included diagnosis of UC, preoperative albumin levels, smoking, and use of C20 mg of Prednisone.	Included a wide range of medical and surgical complications and severity /Small sample size; did not account for difference in resource utilisation	CASP 3*
*Irritable Bowel syndrome*						
Taft et al. [54] USA	1. IBS 2. 243 patients 3. Caucasian (214); non-Hispanic (221); Hispanic (22) *[as reported by authors]* 4. Total mean age 8.7 + 13.5; male (nil); female (209) 5. nil	1. Cross-sectional survey-based 2. Questionnaires 3. Not stated; appears purposive 4. One university-based outpatient gastroenterology clinic, online sources and five private practices	a. ISMI; modified for IBS scale for mental illness a. PSS-IBS c. Clinical data measured IBS subtype, diagnosis duration, symptom duration prior to diagnosis and symptom frequency	Hispanics reported more perceived stigma for personal relationships and healthcare professionals, compared to non-Hispanics, suggesting that there might be cultural differences in IBS-related stigma experience and highlighting the importance of cultural competence in working with Hispanics with IBS.	/Small sample size; generalisability; not powered to detect differences	CEBMa 3*
Wong et al. [56] Malaysia	1. IBS 2. 16 patients 3. Chinese (8); Indian (5); Malay (3) 4. Median age 67 (13.6); male (6); female (10) 5. Nil	1. Pilot observational cohort 2. Cases/telephone interviews 3. Purposive/convenience 4. Dietetic gastroenterology clinic	c. Patient records and prospective telephone interviews measured detailed dietary assessments and self-reported symptom record sheet	Compliance with a low FODMAP diet was poor. For patients who complied (complete/partial) with the diet, symptom improvement was reported as: abdominal pain, abdominal bloating/distension and flatulence. Patients with the IBS-D subtype had the greatest improvement in stool consistency.	/Representation of older and female Malaysians; subjective assessment of IBS symptoms	CEBMa 4*
*Coeliac disease*						
Butterworth et al. [44], UK	1. Coeliac disease 2. 130 patients 3. South Asian (40), White Caucasian (90) 4. Over 16; male (27 White Caucasian, 9 South Asian); female (39 White Caucasian, 12 South Asian) 5. Various (unspecified)	1. Cross-sectional survey-based with case note review 2. Questionnaires; cases 3. Not stated; appears purposive 4. Hospital clinic database	c. Patients’ notes measured small bowel histology and endomysial antibody status at follow-up e. Questionnaire measured experiences of living with coeliac and GFD	Compared to White Caucasians, South Asians managed GFD sub-optimally and needed culturally relevant education. South Asians were less likely to attend dietic clinics, join the Coeliac Society and they were more dissatisfied with information provided by doctors and dieticians compared to White Caucasians; they were dissatisfied with dietic advice. There was a correlation between the perception of a strict GFD and normal or near normal small bowel histology and negative endomysial antibody status at follow-up for White Caucasians.	Generalisation of findings/low questionnaire response due to language or literacy issues	CEBMa 3*

Characteristics and main findings of included studies (n=14) describing study population, design, outcome measures, main findings, strengths/limitations, and quality reported. *CD* Crohn’s Disease, *IBD* Inflammatory Bowel Disease, *UC* Ulcerative Colitis, *SEM* Standard Error of Mean, *HBCS* Hill–Bone Compliance Scale, *QOL* Quality of Life, *SIBDQ* Short Inflammatory Bowel Disease Questionnaire; *TIPS* Trust-in-Physician Scale, *FODMAP* Fermentable Oligosaccharides, Disaccharides, Monosaccharides and Polyols, *GFD* Gluten Free Diet, *IBS* Irritable Bowel Syndrome, *ISMI* Internalized Stigma for Mental Illness, *PSS-IBS* Perceived Stigma Scale for IBS. Overall quality of studies was based on 4* = criteria met well; 3* = criteria moderately met; 2* = poorly met criteria, 1* = criteria not met

### Quality assessment

Based on CASP and CEBMa quality criteria, there were seven good (four star) quality studies [43, 47–49, 51, 53, 56], and seven moderately good (three star) quality studies [44–46, 50, 52, 54, 55].

**Fig. 2:**
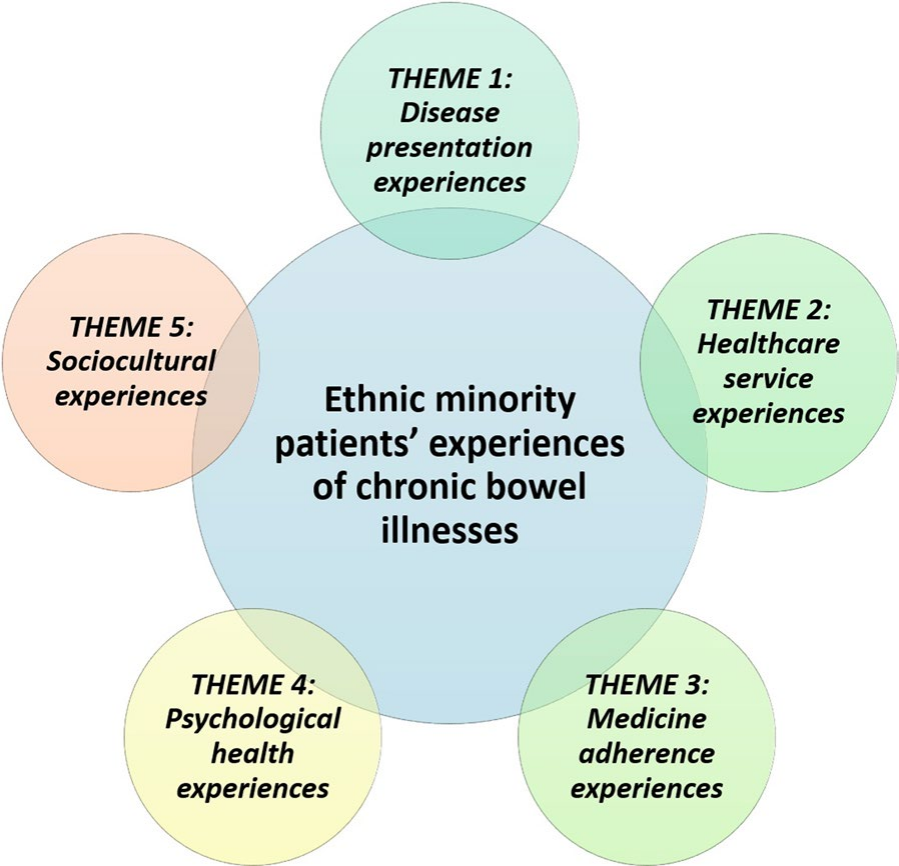
Ethnic minority patients’ experiences of chronic bowel conditions

Narrative synthesis of qualitative and quantitative data produced five broad themes: (1) disease presentation experiences, (2) healthcare service experiences, (3) medicine adherence experiences, (4) psychological health experiences, and (5) sociocultural experiences (Fig. 2).

### THEME 1: Disease presentation experiences

#### Inflammatory Bowel Disease

There were mixed findings on the disease presentation of CD between ethnicities in two US studies [51, 53]. Jackson and Shaukat [51] reported that although the number of annual flare ups and symptom duration before CD diagnosis did not differ, the nature of CD was different in African Americans compared to White patients, where small bowel disease (84% vs. 65%, *p* = 0.03) and small bowel resection (59% vs. 16%, *p* = 0.01) were significantly more prevalent in White patients, though colonic disease (89% vs. 63%, *p* = 0.002) and perirectal fistulae (58% vs. 22%, *p* = 0.001) were significantly higher in African Americans. Straus et al. [53] found that US Black and White patients had similar CD presentations e.g., age of onset. Goodhand et al. [47] reported that 60% of British Bangladeshi participants had significantly more extensive UC disease compared to 33% of White Caucasians (*p* = 0.02), alongside anaemia (*p* =  <0.001) and vitamin D deficiency (*p* =  <0.05).

In relation to IBD diagnosis, Goodhand et al. [47] found that British Bangladeshis were diagnosed with CD significantly more often than with UC, in comparison to White Caucasians (*p* =  <0.01), although there were no significant differences in participant age at diagnosis between these ethnicities (*p* = 0.52). Jackson and Shaukat [51] found that there were no significant differences in the setting in which CD was diagnosed for US African American and White patients [clinic: 31% vs. 61%; hospital: 47% vs. 27%, *p* = ns (not reported)], the number of disease flares (3.69 vs. 3.1 visits per year), and the duration of symptoms before diagnosis (mean duration of disease 3.5 vs. 5.3 years). Alexakis et al. [43] reported that IBD was sometimes misdiagnosed amongst British South Asian and Black participants as tuberculosis, IBS, stress with diarrhoea or psychosomatic disorders.

Two studies found that IBD-related surgeries were similar across ethnicities [46, 53]. Farrukh and Mayberry [46] found that surgery (n = 3 vs. n = 7) and death rates (n = 1 vs. n = 3) were similar for British South Asians and White Europeans. Experiences of post-surgery complications have been reported in two studies [47, 55]. Goodhand et al. [47] found that compared to British White Caucasians, Bangladeshis had significantly later surgery [HR (95% CI) 0.4 (0.2, 0.9), *p* = 0.03], developed more perianal complications [HR (95% CI) 8.6 (1.4, 53.1), *p* = 0.02], and received anti-TNFs earlier [HR (95% CI) 3.0 (1.2, 7.7), *p* = 0.02]. Additionally, Yarur et al. [55] found increased post-operation complications for US Hispanics and non-Hispanics who had equal access to medical aid and follow-up, but this did not reach significance [OR 1.06 (95% CI) 0.48–2.36, *p* = 0.88]. However, postoperative complications were significant factors associated with diagnosis of UC [OR 5.4 (95% CI) 1.67–20.58, *p* = 0.004], preoperative albumin levels [OR 8.2 (95% CI) 2.3–35.5, *p* = 0.001], smoking [OR 15.7 (95% CI) 4.2–72.35, *p* = 0.001], and prednisone use [OR 6.7 (95% CI) 2.15–24.62, *p* = 0.001] [47]. Farrukh and Mayberry [46] found that compared to White Europeans, IBD hospitalisations were more common in South Asians [*p* = ns (not reported)], who also had significantly fewer tests covering wider modalities (t = 2.1, *p* =  <0.02). Additionally, US White participants were significantly more likely to receive multiple doses of infliximab for CD compared to African Americans (34% vs. 11%, *p* = 0.005) [51]. Another study [53] found that IBD-related hospitalisations were similar for US Black and White patients [8.3 (SD 15.5) vs. 10.2 (SD 17.6), *p* = ns (not reported)].

#### Irritable Bowel Syndrome

The selected papers addressing IBS [54, 56] did not focus on experiences relating to disease presentation.

#### Coeliac Disease

Butterworth et al. [44] found that the presentation of coeliac disease (e.g., the morphological recovery of small bowel mucosa to normal/near normal) between British White Caucasians and South Asians was similar, though not statistically significant [75% vs. 77.8%, *p* = ns (not reported)].

### THEME 2: Healthcare service experiences

Several UK and US studies reported the need for better healthcare services for ethnic minority patients [43, 44, 46, 48, 51, 53].

#### Inflammatory Bowel Disease

Attendance at UC follow-up appointments was more common for British White patients compared to other ethnicities; Farrukh and Mayberry [46] found that South Asians were significantly less likely to receive consultant appointments (z = 1.66, *p* =  <0.048) and more likely to be discharged from hospital follow-up (z = -2.3, *p* =  <0.01). Most of the consultants seen by South Asians were European and male. The number of screening colonoscopies offered to White Europeans (43%) was higher compared to South Asians (32%), although this was non-significant [z = 0.9, *p* = ns (not reported)].

Jackson and Shaukat [51] found that US White patients were more likely to seek care for their CD in a clinic setting, whether via primary community physician (1.31 vs. 0.21 visits per year, *p* = 0.001), or secondary (hospital) gastroenterologist care (3.2 vs. 2.3 visits per year, *p* = 0.03). In two IBD studies with British South Asians, participants reported experiencing better medical care and expertise in secondary gastroenterology care compared to primary care [43, 48], though language barriers still existed [48]. Strauss et al. [53] suggested that US ethnic minority groups may have financial constraints in accessing healthcare. White patients were more likely to have health insurance compared to Black patients (92% vs. 85%, *p* = 0.02), who were more likely to be receiving Medicaid (17% vs. 6%, *p* = 0.01), report unreasonable healthcare delays (mean 1.4 vs. 1.3, *p* = 0.01) and have financial difficulties/concerns about affording healthcare (mean 2.8 vs. 2.6, *p* = 0.03), which meant delaying appointments (mean 2.8 vs. 2.6, *p* = 0.02) and travelling to appointment sites (mean 2.8 vs. 2.6, *p* = 0.01). The number of CD-related work absences were also more common amongst Black patients, compared to White patients (*p* =  <0.01).

#### Irritable Bowel Syndrome

Taft et al. [54] found that Hispanic patients with IBS reported more perceived stigma from healthcare providers than Caucasian and non-Hispanic patients (mean 2.30 vs. 1.19, *p* = 0.000).

#### Coeliac Disease

Attendance at coeliac disease follow-up appointments was more common for White patients compared to other ethnicities [44]. Butterworth et al. [44] reported that compared to White Caucasians, South Asians living with coeliac disease were less likely to attend dietician consultations (62.5% vs. 21%, *p* = 0.005), they were significantly dissatisfied with information provided by clinicians and dieticians (8.47% vs. 30%, *p* = 0.03), and dietetic advice (6.35% vs. 30%, *p* = 0.01); reasons for both these experiences were unclear.

### THEME 3: Medicine adherence experiences

Five studies reported experiences of medication use with varied findings [48, 49, 51–53].

#### Inflammatory Bowel Disease

Two studies showed that US White participants with IBD had higher medication adherence compared to African American (77% vs. 49%, *p* = 0.004) [51], and Black minority patients (HBSC: 15.6 vs. 14.0, *p* = 0.0002) [52]. Compared to White patients, African Americans significantly discontinued CD medication due to feeling better (27% vs. 9%, *p* = 0.02), though they knew that this would cause disease flare ups (25% vs. 9%, *p* = 0.036) [51], and IBD medicine adherence amongst Black patients was significantly related to greater trust in physicians (R =  −0.30, *p* =  < 0.0001), increasing age (R = −0.19, *p* = 0.01) and worsening health-related quality of life (QOL) (R = −0.18, *p* = 0.01) [52]. In two other studies, similar medication adherence was reported for the majority of British South Asians and White Europeans with IBD [48], and between US Black and White participants with CD [92% vs. 88%, *p* = ns (not reported)] [53]. However, many South Asians also used complementary and alternative medication (CAM) alongside prescribed medication (e.g., Ayurvedic medicine and Isabgol), and some consulted faith healers [48]. South Asian Muslims living in the UK had no clear information on whether they could use medication during the fasting hours of Ramadan, which influenced adherence [49].

#### Irritable Bowel Syndrome and Coeliac Disease

Medication adherence was not addressed in the selected articles which focussed on stigma in IBS [54], dietary aspects in IBS [56], and GFD in coeliac disease [44].

### THEME 4: Psychological health experiences

Three of the selected papers reported on the psychological wellbeing of patients with IBD and IBS [45, 48, 54].

#### Inflammatory Bowel Disease

Two UK-based studies reported experiences of IBD-related anxiety amongst South Asians [45, 48]. Provision of patient information booklets translated into common South Asian languages (Hindi, Gujarati, Punjabi) reduced or had no effect on IBD-related anxiety in 66% of participants, whilst 33% reported increased levels of anxiety, although the sample size was small (N = 56) [45]. Mukherjee et al. [48] found that due to the fear of becoming symptomatic, other IBD-related emotional experiences (e.g., depression and feeling low) played a role in inhibiting engagement of social activities during asymptomatic periods.

#### Irritable Bowel Syndrome

Taft et al. [54] reported that higher levels of anxiety were linked to higher levels of perceived stigma, and that Hispanic participants reported higher levels of perceived stigma from personal relationships and from healthcare providers when compared with non-Hispanic participants. This suggests that Hispanic patients with IBS experience higher levels of disease-related anxiety than non-Hispanic patients, although it is not explicitly stated. Disease-related psychological impact was not addressed in the single paper reporting the potential for a low FODMAP diet to benefit people from ethnic minority groups with IBS [56].

#### Coeliac Disease

The single paper addressing GFD in coeliac disease [44] did not report any data linked to disease-related psychological impact.

### THEME 5: Sociocultural experiences

Sociocultural aspects were described in four sub-themes relating to health-literacy and culturally relevant information, diet, social support, and religion.

#### Sub-theme: Health literacy about bowel conditions and need for culturally relevant information

***Inflammatory Bowel Disease***

The need for culturally relevant information and education for British South Asians living with IBD was identified by three studies [43, 45, 48]. Low health literacy about IBD amongst British South Asians and the wider community was reported by two studies [48, 49]. Mukherjee et al. [48] found that the South Asian community had difficulty understanding IBD because there was no substitute word for ‘Crohn’s’ in some languages and there were different connotations of the word ‘disease’ as in the label ‘inflammatory bowel disease’—for example, disease may also infer infectious or life-threatening illnesses. Communication about bowel symptoms with other people was perceived as private due to factors such as embarrassment, stigma (including concerns about marriageability and conceiving children) and conflict around cultural expectations, such as gender roles for women’s ability to manage childcare and housework, and men’s ability to be a provider.

Mukherjee et al. [48] also found that South Asians had barriers in using the online Crohn’s and Colitis UK (charity) website due to language factors, IT literacy and culturally appropriate venues where participants would not stand out as the only South Asian in a group. Nash et al. [49] reported that younger British South Asians who were proficient in English were able to access and understand information, but there was little culturally relevant information for parents who spoke English as a second language. Additionally, Jackson and Shaukat [51] reported that both US White and African American participants felt equally informed about CD. Conroy and Mayberry [37] described the difficulties of even recruiting UC patients to their study due to communication difficulties and lack of resources in relevant languages. They concluded that greater detail may be needed to make the content of information leaflets more culturally relevant.

***Irritable Bowel Syndrome***

Wong et al. [56] focussed on compliance with a low Fermentable Oligosaccharides, Disaccharides, Monosaccharides and Polyols (FODMAP) diet to improve IBS symptoms. They do not report any data indicating the likely influences on the low compliance rate (50% complete compliance over a 6-week programme), although cultural influences and limited knowledge amongst patients are addressed in the Discussion section.

***Coeliac Disease***

Butterworth et al. [44] report that factors correlated with stated compliance to a GFD in White Caucasians do not correlate with stated compliance in South Asian patients, including understanding of food labelling [2.13 (OR1.08–4.17) vs. 1.21 (OR 034–4.34)] and receiving a detailed explanation of coeliac disease from their physician [2.04 (OR 1.16–3.57) vs. 1.59 (OR 0.36–7.14)]. South Asians were more dissatisfied than White Caucasians with information provided by their physician (30% vs. 8.47%, *p* = 0.03) and with dietetic advice (30% vs. 6.35%, *p* = 0.01). The authors concluded that verbal and written information about coeliac disease and a GFD, provided in appropriate languages, are necessary to increase health literacy and enhance compliance with treatment.

***Sub-theme: Diet***

Five out of fifteen studies focussed on experiences of diet in relation to IBD, IBS and coeliac disease [43, 44, 49, 50, 56].

***Inflammatory Bowel Disease***

Damas et al. [50] found that US Hispanics, of which a majority had adapted to bicultural acculturation [IBD 17 (58.6%); controls 21 (72.4%), χ^2^ (3, n = 58) = 3.31, *p* = 0.35], changed their diet after migration with no active gastrointestinal symptoms at the time of migration, though this did not reach significance [IBD 72.4%; controls 57.1%, χ^2^ (1, n = 57) = 1.46, *p* = 0.23). Hispanic participants reported that they developed poor dietary intake (e.g., lower than recommended consumption of vegetables, legumes, whole grains, and sea plant protein), irrespective of whether they had IBD or not [mean IBD 53.8 (SD 13.9); controls 56.5 (SD 10.5), t(55) =  −0.81, *p* = 0.42, d = 0.22]. Although, IBD patients reported lower intake for total fruit [mean IBD 2.5 (SD 2.3); controls 3.7 (SD 1.7), *p* = 0.02], whole fruit [mean IBD 2.5 (SD 2.4); controls 3.8 (SD 2.0), *p* = 0.02], and total dairy [mean IBD 3.1 (SD 3.4); controls 5.7 (SD 3.5), *p* = 0.01], but higher consumption of sodium [mean IBD 2.0 (SD 2.6); controls 3.5 (SD 3.1), t(55) =  −1.9, *p* = 0.06, d = 0.52], and refined grains [mean IBD 5.5 (SD 3.8); controls 7.6 (SD 3.3), t(55)  =  −2.2, *p* = 0.03, d = 0.59]. Participants with IBD also met recommended minimal consumption of empty calories such as alcohol, solid fats and added sugars [mean IBD 17.5 (SD 3.9); controls 13.2 (SD 5.2), t(55) = 3.56, *p* =  < 0.01, d = 0.93].

Three UK-based studies showed that self-management of diet in IBD revolved around avoidance of certain food to reduce symptoms [43, 48, 49]. However, sometimes there was a conflict and struggle with cultural norms where food was shared (for example, spicy food, religiously blessed food, family functions and women living with in-laws), which meant that patients had daily practical and emotional challenges, including anxiety, social exclusion, a sense of loss, social pressure to eat, difficulty getting others to accept their chosen diet and guilt around becoming a burden [43, 48, 49]. Those participants who attended social events, sometimes compromised by bringing separate packed food, or had others prepare separate meals for them [43]. One study [49] found that little IBD awareness amongst elders also caused tensions in understanding the chosen diet of young people, who found it was difficult to decline requests of elders encouraging them to eat certain foods that were perceived to be healthy. Conflict led to practical and emotional toll such as hurtful comments about appearance and weight.

***Irritable Bowel Syndrome***

A Malaysian study [48] found poor compliance with a low FODMAP diet in Chinese, Indian and Malays. However, those who completely or partially complied with the diet improved IBS-related bowel symptoms of flatulence (87.5%), bloating/distension (70%) and abdominal pain (60%). Limited access to low FODMAP food items and poor labelling of FODMAP content in Asian foods were reported as contributing to low adherence in the non-compliant group.

***Coeliac Disease***

Butterworth et al. [44] found that more British White Caucasians living with coeliac disease significantly reported that they never ingested gluten (*p* = 0.04), or ingested gluten less than once a month compared to South Asians (*p* = 0.03), suggesting that the management of GFD in South Asians may need to be different to White Caucasians, who were more likely to understand food labelling, had access to gluten-free products and were members of the Coeliac Society (charity).

#### Sub-theme: Social support

***Inflammatory Bowel Disease***

Social support for South Asians living with IBD was limited [43, 48, 49] due to language barriers, lack of culturally relevant information, relying on information from lay sources, and for younger patients, difficulties of explaining or censoring information to parents to avoid burdening them, such as not mentioning the chronic nature of IBD [43, 49]. Some South Asian parents believed that IBD was related to ulcers or poor diet and did not know whether they should inform their child’s school [43]. Disruption to education was also reported by various ethnic minority groups [43, 49] who reported a lack of integrated IBD understanding and care for young people at schools, which could result in bullying [43].

***Irritable Bowel Syndrome***

Taft et al. [46] found that compared to non-Hispanics, US Hispanics reported significantly more perceived IBS-related stigma for personal relationships [mean 2.90 vs. 1.67, t(196) = 9.24, *p* = 0.000]. Items in the Perceived Stigma Score-IBS demonstrate higher scores (greater perceived stigma; maximum score per sub-scale 5.0) from significant others not having enough knowledge about IBS (3.2 ± 1.3), not taking the person with IBS seriously (2.5 ± 1.3), not being interested in hearing about IBS (2.8 ± 1.4), although the authors do not report Hispanic and non-Hispanic data separately.

***Coeliac Disease***

Fewer South Asians than White Caucasians accessed social support via membership of the Coeliac Society (53.4% vs. 79%, *p* = 0.02); membership was reported as one of the factors to influence compliance with a GFD [44].

#### Sub-theme: Religious self-management

***Inflammatory Bowel Disease***

Three studies found that religious coping was important for self-managing IBD, particularly for British South Asian Muslims. Religious actions had a calming effect, such as helping participants understand why they were experiencing illness, dealing with pain, and believing that IBD was a test from God [43, 48, 49]. Support from religious leaders, including empathy and leaflets on religious guidance during Ramadan, was also noted as beneficial for some Muslims [43]. Additionally, managing symptoms was important to participate in Islamic worship [43, 48, 49]; fear of incontinence and anticipated bowel movements created anxieties for Muslims around maintaining ablution (an Islamic ritual that forms the basis of performing various types of worship such as prayer) [43, 48], preserving a clean place of worship and avoiding interruptions to prayer [43].

***Irritable Bowel Syndrome and Coeliac Disease***

None of the papers addressing IBS [54, 56] or coeliac disease [44] reported data addressing religious influence as these quantitative studies addressed measuring stigma, and the effects of specific diets on these diseases.

## Discussion

Fourteen studies were identified, with a mixture of good and moderately good quality, which explored diverse experiences of ethnic minority patients from the UK, US, and Malaysia, living with IBD, IBS, and coeliac disease. Culturally relevant IBD and coeliac disease information/education was needed for British South Asians due to low awareness, language and literacy barriers, and illness perceptions—including understanding IBD to be a private matter that should not be discussed openly. Muslim South Asians living with IBD used religious self-management to understand illness experiences and had difficulty in managing symptoms (e.g., fear/risk of incontinence) to fulfil religious activities. British South Asian and Black individuals had problems with IBD diagnosis and misdiagnosis. Ethnic minority populations across countries and illnesses experienced poor dietary intake, difficulties adhering to a GFD, cultural conflict in self-managing one’s diet (such as avoiding spicy food), increased IBD-related anxiety, and received poor-quality healthcare services particularly in primary care in the UK. Mixed findings on experiences of disease presentation showed that the nature of conditions was sometimes similar (for example, coeliac disease in South Asians), and at times extensive (such as UC in Bangladeshis), compared to the White population. Experiences of medicine adherence varied across different studies; some ethnic minority groups with IBD had poorer medicine adherence (e.g., US Black and African American patients) and some had similar medicine adherence (e.g., US Black patients), compared to White patients. Some South Asians with IBD also used CAM alongside medication.

Low IBD awareness amongst South Asians (and relevant others), generally due to language barriers [48, 49], has previously been reported for other chronic illnesses such as cancer and cancer-related services e.g., colorectal screening [57, 58], and may indicate generational differences, including older generations for whom English is a second language. Understanding of illnesses may need to be facilitated by culturally relevant information [43–45, 48, 49], cultivated with linguistic considerations including the use and meaning of non-equitable terms—such as ‘Crohn’s’ ‘Disease’—in other languages [48]. What constitutes as an illness may differ cross-culturally and from Western descriptions [21]. The perception, expression and management of an illness and its symptoms (e.g., pain) may be shaped by different cultural influences (e.g., belief systems), psychosocial factors and relationship structures [21, 59]. For instance, Indians can conceptualise IBS in terms of emotions such as anxiety and depression, hence why there may often be a diagnosis stigma related to psychological instability in this community. In comparison, family relationships; a Mexican cultural value may mean emotions experienced in relationships such as stress are attributed to IBS. While Chinese individuals may appraise their connection with their environment, therefore during symptomatic periods of IBS they may express a sense of imbalance with the environment and a need to re-balance through self-management [59].

Using diet to manage symptoms [43, 48, 49] has been widely reported such as avoiding spicy food [22, 26] and dining outside the home [26], although this review found that poor understanding of dietary choices of young people with IBD were often not aligned with traditional norms and expectations of parents, in-laws, or extended family, creating psychological issues (e.g., anxiety) and generational conflict [43, 48, 49]. Dietary changes due to migration/acculturation lead to poor meal intakes for Hispanics living with IBD [50], and is supported by previous studies; in Norway, Pakistani and Sri Lankan migrants reduced intake of beans and lentils [60], and Pakistani women increased dairy intake [61]. British South Asians had higher energy and fat intake and lower carbohydrates [62], while Canadian South Asian children had higher intake of fat and refined sugars, and lower intake of fresh fruits and vegetables compared to their parents and grandparents [17]. Literacy issues can also have an impact on the efficacy of recommended dietary changes (such as understanding food content, acceptability and access to proposed food as with GFD, understanding what constitutes as high and low FODMAP diets) [63–65], as found in this review—South Asians with coeliac disease did not understand food labelling [44].

Stigma related to IBS and IBD were widespread in relation to gender expectations (e.g., conceiving children), and personal relationships [48, 54], although such stigma has also been reported in other ethnic groups [23, 66, 67]. Discretion in discussing bowel symptoms were also relevant to Pakistani women with urine incontinence [48, 68]. Anxieties around IBD symptoms (e.g., fear of incontinence and bowel movements) on managing daily religious duties based on physical purification for Muslim South Asians [43, 48, 49] has been previously reported with IBS [69], colostomy procedures [70, 71], and in urinary incontinence [22, 68]. Since certain bowel symptoms may nullify the state of purification, therefore during symptom flare-ups there may be additional self-management challenges due to the repeated need for ablution and needing to be near washing facilities [72]; these challenges may be heightened during religious months such as Ramadan and the Hajj pilgrimage [70, 71]. Guidance in using medication during Ramadan could be addressed in future interventions [49]. Religious actions were noted to have positive impact on experiences of IBD in our review [43, 48, 49], however other studies revealed that surgical interventions may instead reduce QOL [70, 71, 73]. One literature review [71] of stoma patients found that perceptions of symptoms (e.g., uncleanliness) had a negative impact on psychological, religious, and spiritual well-being, since patients were restricted in fully immersing themselves in religious activities after surgery, for instance participation in congregational mosque prayers and limiting the frequency or complete cessation of prayer. Fear of damaging the stoma was mentioned as a contributing factor to ceasing fasting in Ramadan, though it is medically safe [70, 71].

Evidence of generally poor health outcomes for ethnic minority groups in the UK and US [43, 44, 46, 47, 51, 55] suggest the need for a deeper consideration of existing inequalities in healthcare services [22, 43, 44, 46, 48, 51, 53]. Earlier CD studies have also found high unscheduled hospitalisations for US Asians and emergency visits for African Americans [2]. Access disparities in IBD treatment have been found to vary across UK regions, where British South Asians and Eastern Europeans were significantly less likely to be hospitalised compared to White individuals, while in other areas compared to British White people, Afro-Caribbean patients received significantly less treatment [74]. Findings of this study should be taken with the caveat that summarising data across countries may overlook the interactions created between an individual and their environment, for instance socioeconomic factors in accessing medical care may be more relevant to the US due to Medicaid [2], as found in this review [53]. In the UK, reasons for such disparities are more ambiguous [75], though it may include discrimination, language differences, restrictions in choosing the gender of healthcare professionals [76], and limited awareness of available services [75]. Inequitable provisions to manage language diversity, as found in gastroenterology services in this review [48], has previously been found to influence communication with healthcare professionals [68, 75].

## Limitations

To our knowledge, this has been one of the few studies reviewing literature on the experiences of ethnic minority patients’ living with chronic bowel illnesses. At full text screening, we excluded potentially relevant papers on cancer screening for bowel illnesses since they included participants who may not have pre-existing chronic bowel conditions; however, these papers may have been useful in understanding attitudes of the wider community and merit separate exploration. Caution should be taken when considering the findings of the review, as some studies did not account for the diversity within a population e.g., defining ethnicity as ‘Blacks’ and ‘Hispanics’, implying that researcher approach to defining ethnicity needs to be reported. One finding may be relevant for an ethnic group in one country but not others [2]. We included two studies that did not fully specify the ethnic group of participants from Hispanic backgrounds [54, 55]; for example, Yarur et al. [55] described participants as those from Latin American descent, and Spanish and Portuguese origins, and Caribbean, Black or Other. We accepted the search term ‘Hispanic’ as a baseline descriptor of ethnicity and therefore included these studies. We acknowledge that this review includes different studies with several pathological conditions and ethnic groups, which may limit the wider application of conclusions.

## Conclusions

This review has explored experiences of ethnic minority patients living with bowel illnesses across contexts and has identified that significant gaps remain in unearthing the experience and perspective of individuals who may not be able to speak English easily. Further qualitative work is needed to understand the cultural sensitivity of such experiences, and to build on extant preliminary data on experiences of psychological health, social support, and religious self-management. A generational and religious lens in understanding contextual experiences of ethnic minority groups may be necessary to understand, for example, cultural conflict in relation to diet. There is also a need to culturally tailor information for patients and those who support them, by addressing language and literacy barriers in healthcare services. More research is needed to understand and test the acceptability and feasibility of tailored information. Inequalities in healthcare services and health outcomes suggest multilevel contextual factors may be at play, specific to the countries in question. Future research with ethnic minority populations experiencing other bowel-related conditions such as stoma and anterior resection syndrome following treatment for rectal cancer, is required.

## Supplementary Information

**Additional file 1: Appendix S1.** Detailed search strategy. Dataset. FigShare 2021: https://doi.org/10.6084/m9.figshare.13110608.

## Data Availability

All included papers are published. The data that support the findings of this review are available from the corresponding author upon reasonable request.

## Abbreviations

CAM: Complementary and Alternative Medication
CASP: Critical Appraisals Skills Programme
CD: Crohn’s Disease
CEBMa: Centre for Evidence-Based Management Critical Appraisal
FODMAP: Fermentable Oligosaccharides, Disaccharides, Monosaccharides and Polyols
GFD: Gluten Free Diet
IBD: Inflammatory Bowel Disease
IBS: Irritable Bowel Disease
QOL: Quality of Life
SPIDER: Sample, Phenomenon of Interest, Design, Evaluation and Research type
UC: Ulcerative Colitis
