# Supplementary material for: Experiences of ethnic minority patients who are living with a primary chronic bowel condition: a systematic scoping review with narrative synthesis
Source: BMC Gastroenterol. 2021 Aug 18;21:322. doi: 10.1186/s12876-021-01857-8 (PMC8371833; doi:10.1186/s12876-021-01857-8)
Supplement: Supplementary file 1 — Additional file 1: Appendix S1. Detailed search strategy. Dataset. FigShare 2021: https://doi.org/10.6084/m9.figshare.13110608. [file 12876_2021_1857_MOESM1_ESM.docx]

### Supplementary file 1: Detailed search strategy

*Basic search strategy:* Sample **AND** Phenomenon of interest **AND** Design **OR** Evaluation **OR** Research type **NOT** Genetics (FILTERS: 2000; English; Humans)

### All searches in (title/abstract)

### Exemplar of search strategy on Medline database (Date searched, 09/12/19)

| **Search** | **Search** | **Items found** |
| --- | --- | --- |
| #1 | Ethnic* OR Minorit | 146905 |
| #2 | ((Ethnic* OR Minorit*)) AND (Inflammatory bowel disease OR Crohn OR Ulcerative colitis) | 687 |
| #3 | ((Ethnic* OR Minorit*)) AND (bowel disease OR Crohn OR Ulcerative colitis) | 3587 |
| #4 | ((Ethnic* OR Minorit*)) AND (bowel OR Crohn OR Ulcerative colitis) | 1434 |
| #5 | (((Ethnic* OR Minorit*)) AND (Inflammatory bowel disease OR Crohn OR Ulcerative colitis)) AND (Interview OR Focus group OR Survey) | 115 |
| #6 | (((Ethnic* OR Minorit*)) AND (Inflammatory bowel disease OR Crohn OR Ulcerative colitis)) AND (Interview OR Focus OR Survey) | 120 |
| #7 | ((((Ethnic* OR Minorit*)) AND (Inflammatory bowel disease OR Crohn OR Ulcerative colitis)) AND (Interview OR Focus group OR Survey)) OR Experien* | 604121 |
| #8 | ((((Ethnic* OR Minorit*)) AND (Inflammatory bowel disease OR Crohn OR Ulcerative colitis)) AND (Interview OR Focus group OR Survey)) OR Experience OR Experiential | 354615 |
| #9 | ((((Ethnic* OR Minorit*)) AND (Inflammatory bowel disease OR Crohn OR Ulcerative colitis)) AND (Interview OR Focus group OR Survey)) AND Experience OR Experiential | 5361 |
| #10 | ((((Ethnic* OR Minorit*)) AND (Inflammatory bowel disease OR Crohn OR Ulcerative colitis)) AND (Interview OR Focus group OR Survey)) AND Experien* | 7 |
| #11 | (((((Ethnic* OR Minorit*)) AND (Inflammatory bowel disease OR Crohn OR Ulcerative colitis)) AND (Interview OR Focus group OR Survey)) AND Experien*) OR (Qualitative OR Quantitative OR Mixed methods) | 405851 |
| #12 | (((((Ethnic* OR Minorit*)) AND (Inflammatory bowel disease OR Crohn OR Ulcerative colitis)) AND (Interview OR Focus group OR Survey)) AND Experien*) AND (Qualitative OR Quantitative OR Mixed methods) | 1 |
| #13 | (((((Ethnic* OR Minorit*)) AND (Inflammatory bowel disease OR Crohn OR Ulcerative colitis)) AND (Interview OR Focus group OR Survey)) AND Experien*) AND (Qualitative OR Quantitative) | 1 |
| #14 | (((((Ethnic* OR Minorit*)) AND (Inflammatory bowel disease OR Crohn OR Ulcerative colitis)) AND (Interview OR Focus group OR Survey)) AND Experien*) OR (Qualitative OR Quantitative) | 337200 |
| #15 | (((((Ethnic* OR Minorit*)) AND (Inflammatory bowel disease OR Crohn OR Ulcerative colitis)) AND (Interview OR Focus group OR Survey OR Qualitative OR Quantitative)) AND (Experien*) | 7 |
| #16 | (((((Ethnic* OR Minorit*)) AND (Inflammatory bowel disease OR Crohn OR Ulcerative colitis)) AND (Interview OR Focus group OR Survey) OR (Qualitative OR Quantitative) AND (Experien*) | 53134 |
